# Functional microorganisms in Baijiu Daqu: Research progress and fortification strategy for application

**DOI:** 10.3389/fmicb.2023.1119675

**Published:** 2023-01-27

**Authors:** Haideng Li, Shengyuan Liu, Yanbo Liu, Ming Hui, Chunmei Pan

**Affiliations:** ^1^College of Biological Engineering, Henan University of Technology, Henan, Zhengzhou, China; ^2^College of Food and Biological Engineering (Liquor College), Henan University of Animal Husbandry and Economy, Zhengzhou, Henan, China; ^3^International Education College, Henan Agricultural University, Zhengzhou, Henan, China

**Keywords:** Daqu, Baijiu, functional microorganisms, microbiomics, fortification and regulation

## Abstract

Daqu is a saccharifying and fermenting starter in the production of Chinese Baijiu; its quality directly affects the quality of Baijiu. The production of Daqu is highly environment-dependent, and after long-term natural domestication, it is rich in a wide variety of microorganisms with a stable composition, which provide complex and diverse enzymes and flavor (precursor) substances and microbiota for Jiupei (Fermented grains) fermentation. However, inoculation with a relatively stable microbial community can lead to a certain upper limit or deficiencies of the physicochemical properties (e.g., saccharification capacity, esterification capacity) of the Daqu and affect the functional expression and aroma formation of the Daqu. Targeted improvement of this problem can be proposed by selecting functional microorganisms to fortify the production of Daqu. This review introduced the isolation, screening, identification and functional characteristics of culture-dependent functional microorganisms in Baijiu-brewing, the core functional microbiota community of Daqu, and the related research progress of functional microorganisms fortified Daqu, and summarized the fortifying strategies of functional microorganisms, aiming to further deepen the application of functional microorganisms fortification in Daqu fermentation and provide ideas for the flavor regulation and quality control of Baijiu.

## 1. Introduction

As one of the six distilled liquors in the world, Chinese Baijiu is unique in that it uses a strategy of fermentation of raw materials while saccharifying and fermenting (Xu et al., 2017; He G. et al., 2019). Daqu is an essential starter for Chinese Baijiu, as it is rich in microbial communities, functional enzyme systems, and flavor precursors, and plays an important role in the initiation and smooth progress of Jiupei fermentation (Zheng et al., 2011; Wang B. W. et al., 2018). Microbiota from nature is naturally inoculated and assembled into Daqu’s open fermentation process, so it is abundant in bacteria, molds, yeasts, actinomycetes, and other microorganisms (Xu et al., 2017). As the most important microorganism in the brewing process of Baijiu, bacteria play a significant role in enhancing the flavor and quality of Baijiu and have always been the focus of research and hot spots (Du H. et al., 2021). As a class of brew microorganisms, yeast is not only the leading force in the alcoholic fermentation process of Baijiu, but also its aroma production performance is crucial to the quality and characteristics of different flavored Baijiu (Wang D. Q. et al., 2019). Mold in the process of fermentation of the Daqu produces protease, lipase, glycosylase, cellulase, and other rich and diverse enzyme system, with the ability of aldolization, esterification, etc., and the growth of mycelium is conducive to water volatilization and material utilization in the heart of the Daqu and has a vital role in the maturation of the Daqu fermentation (Li et al., 2019; Tang et al., 2022). Actinomyces have a variety of metabolic activities, including hydrolysis of starch, cellulose, protein, and pectin in raw grains, and the production of various secondary metabolites (Chen et al., 2022).

However, when Daqu is naturally enriched and inoculated with various microorganisms in the environment, in addition to assembling beneficial bacteria, bacteria harmful to Baijiu-brewing will inevitably intermingle, resulting in unstable quality, low yield, and high production cost of Baijiu production (Ke et al., 2019). Furthermore, due to environmental dependence, there are certain constraints on the inherent property of Daqu, such as the upper limit of enzyme activity (Zheng et al., 2015; Liu J. J. et al., 2018; Ma et al., 2022). In the early days, to solve such problems, scholars and breweries usually used functional microorganisms isolated from the brewing environment in the manufacture of Daqu or the development of compound bacterial agents to improve the structure and physicochemical properties of the Daqu microbial community and enhance the aromatic substances and flavor precursors of Daqu, thereby improving the yield and quality of the original Baijiu. The reason for choosing *in-situ* fortification is, firstly, to ensure the role of the Daqu in the brewing of Baijiu and to emphasize the innovation based on the traditional process, and secondly, the functional strain fortified Daqu can be used as a stable and effective carrier for the preservation of crude enzymes, microbiota and aroma substances. For example, adding *Saccharomyces cerevisiae* to Daqu can increase the content of nitrile enzyme and significantly degrade the content of cyanide in Daqu (Shen et al., 2021). Inoculation with *Bacillus velezensis* and *Bacillus subtilis* could improve the flavor profile of Daqu and significantly improve the contents of Tetramethyl pyrazine and phenyl ethanol (He G. et al., 2019). However, due to the limitation of research methods, the scientific mechanism of the consequence of functional microorganisms’ fortification on the Daqu is not clear, and often the fortification of functional microorganisms does not achieve the expected effect or lead to the degradation of the quality of Daqu.

In recent years, with the rapid development of ecological fermentation technology, researchers have reconstructed the fermentation microbiota, reorganized the core functional microbiota, and controlled the fermentation process to control the microbiota to produce target products by adjusting the microbiota inoculation ratio (Du H. et al., 2021). Studies have shown that the most effective way to control the microbial community of the mature Daqu is by directly adding microorganisms to change the initial microbiota (Wang et al., 2017). Yet, the key to the realization of the ecological fermentation of Daqu lies in the composition and succession of the microbial community and the analysis of metabolic characteristics of functional microorganisms. Only by clarifying the regulation of different microorganisms on Daqu flavor and physical and chemical indicators, can we further reconstruct fermentation microbiota or construct synthetic microbiota based on *in situ* system (Kong et al., 2014). Among them, the identification of core functional microorganisms is the primary condition for controlling fermentation. Presently, the core functional microorganisms in the fermentation process of Daqu have been identified based on metagenomics and other multi-omics approaches as well as statistical analysis methods such as partial least squares and Pearson correlation analysis. There has been some research, and under this premise, it is helpful to guide the screening of functional microorganisms.

Based on this, this paper systematically summarized the screening, identification, and functional characteristics of culture-dependent Baijiu-brewing functional microorganisms, the core microbial community in the fermentation process of Daqu, and the research progress of functional microorganisms fortified Daqu. Thinking and looking forward to the significance and effect of functional microbial community analysis, pure species isolation and screening, and microbial community recombination on flavor regulation and quality control of Daqu, which has far-reaching significance for the healthy and standardized development of Baijiu.

## 2. Definition and classification of Baijiu Daqu

**Figure 1:**
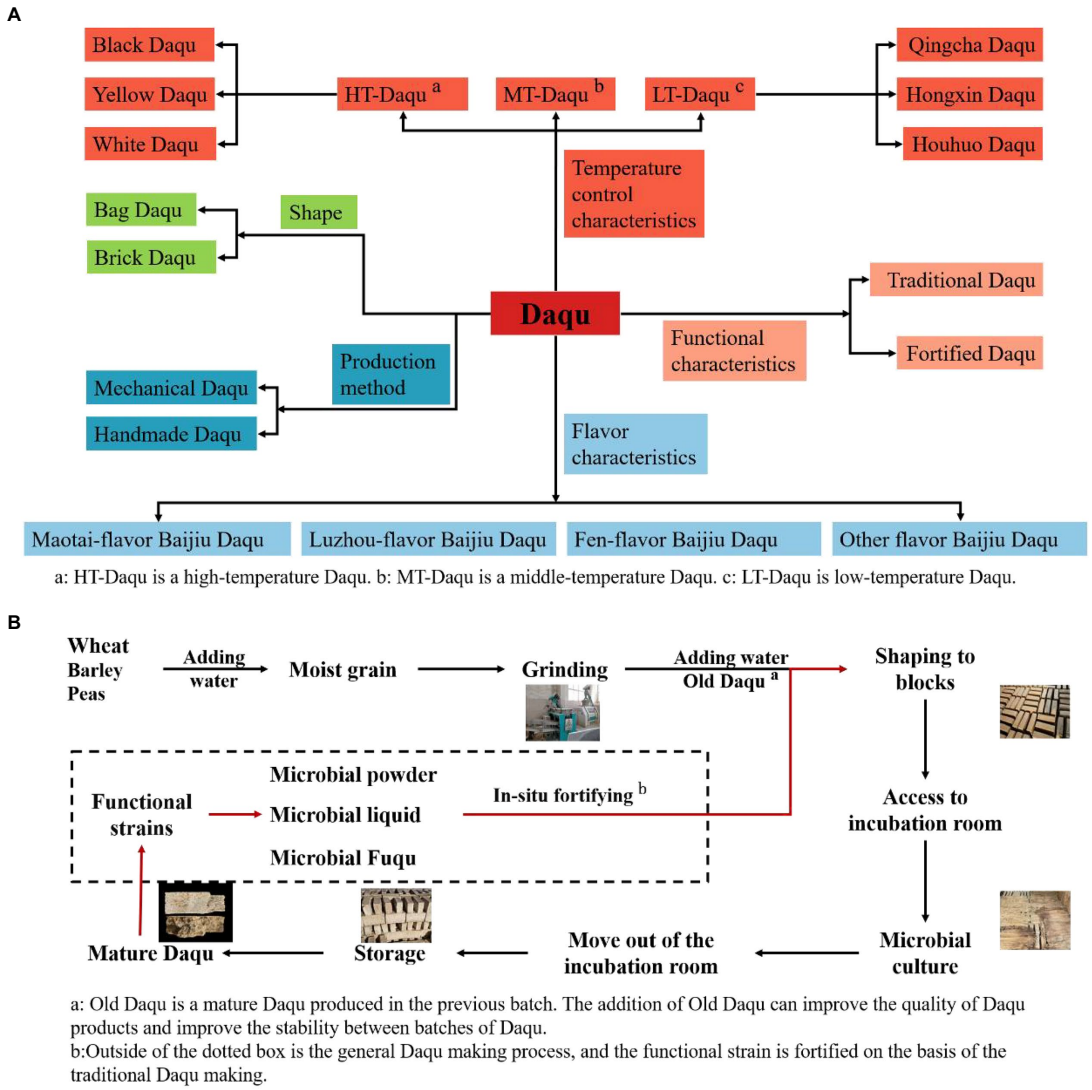
**(A)** Classification of Daqu according to different characteristics. **(B)** Sketch of the fortified Daqu production process.

Daqu is one of the Chinese Baijiu Jiuqus (starters). The main ingredient is raw wheat, supplemented by a percentage of barley and peas (Kang et al., 2022). The raw materials are moistened, grinder, and shaped into blocks and then sent to the incubation room to be naturally inoculated with environmental microorganisms for fermentation, and the finished product is named Daqu because of its shape (Zheng et al., 2011). The fermentation process of Daqu is open, so the enriched microbial community is complex and diverse. Microorganisms interact and evolve in Daqu to form a specific stable microbial community. Microbiota produces a variety of enzymes that play a vital role in the saccharification, alcoholic fermentation and flavor formation of starchy raw materials. Unlike Xiaoqu and Fuqu, Daqu is used as a starter to produce most of China’s famous Baijiu, such as Moutai, Wuliangye, Fenjiu, etc. In the Daqu type division, mainly in Daqu fermentation maximum temperature (High-temperature Daqu, Middle-temperature Daqu, Low-temperature Daqu), flavor characteristics (Maotai-flavor Baijiu Daqu, Luzhou-flavor Baijiu Daqu, Fen-flavor Baijiu Daqu, etc.), shape (Bag Daqu, Brick Daqu), production methods (Mechanical Daqu, Handmade Daqu), functional characteristics (Traditional Daqu, Fortified Daqu) to divide (Zheng et al., 2018; He G. et al., 2019; Zuo et al., 2020; Kang et al., 2022; Figure 1A).

The above classification has the potential for further. For example, High-temperature Daqu due to early fermentation temperature fluctuations will lead to the final Daqu color being different, divided into Black Daqu, Yellow Daqu, and White Daqu (Shi et al., 2022). Low-temperature Daqu was controlled at different temperatures during the fermentation stage to form three kinds of Daqu: Qingcha Daqu, Hongxin Daqu, and Houhuo Daqu (Cai et al., 2021). It seems that naming only focuses on the morphological differences, but it essentially reflects the changes in the metabolism of the microbial community caused by temperature changes. Each Daqu has its unique functional characteristics.

Yet, due to differences in the natural environment, there are often significant differences in the types, numbers, and proportions of microbiota in the Daqu from different geographical environments (Zheng et al., 2015; Ma et al., 2022). This difference is manifested in two aspects: firstly, the difference in the microbial community leads to a slight difference in flavor formation, and such influence is positive in a certain sense, creating a different sensory experience of Baijiu; secondly, the difference in the microbial community leads to defects in the physicochemical properties (such as esterification power and saccharification power) of Daqu, which to a certain extent directly affects the Baijiu wine quality and production cost.

The Daqu and Jiupei solid-state fermentation prohibits the addition of exogenous non-fermentative substances, such as the direct addition of various enzymes. In response to the adverse effects of differences in the microbial community, researchers improved the quality of Daqu by making fortified Daqu in the 1980s. Fortified Daqu improves quality and fermentation characteristics by adding artificially cultured pure functional microorganisms based on traditional Daqu production (Li W. W. et al., 2020). The production process of the Fortified Daqu is shown in Figure 1B.

## 3. Baijiu-brewing functional microorganisms

### 3.1. Definition and research methods of functional microorganisms (microbiota)

Baijiu fermentation is a complex natural fermentation system. The microorganisms involved in fermentation mainly come from Daqu microecology and environmental microecology, and the population and metabolism are complex, thus providing complex and diverse enzyme systems and flavor (precursor) substances for Baijiu fermentation, and giving Baijiu unique taste and flavor (Xiao et al., 2021). Among them, high-yielding enzymes (such as amylase, protease, cellulase, esterase, glucase, tannase, xylanase, pectinase, oxidoreductase, lyase, isomerase, etc) and aroma substances (such as ethyl caproate, ethyl acetate, ethyl butyrate, ethyl valerate, ethyl octanoate, 1-propanol, acetic acid, 2,3,5-Trimethylpyrazine and 2,3,5,6-tetramethylpyrazine, etc) were widely defined as Baijiu-brewing functional microorganisms (Zeng, 2016; Song et al., 2017; Ma et al., 2022; Zhu et al., 2022).

In the early stage, studies on functional microorganisms mainly focused on isolation and purification by traditional culture-dependent methods, identification by molecular biology techniques, enzyme activity determination, metabolic profiling and characterization of microorganisms with alcohol, aroma and enzyme production characteristics (Xing et al., 2018; Wu et al., 2021; Liu Y. B. et al., 2022). Compared with rapid and high-throughput microbial community analysis such as polymerase chain reaction-denatured gradient gel electrophoresis (PCR-DGGE) analysis of DNA extracts, pyrosequencing, and clonal library sequencing, the traditional culture-dependent method has the obvious advantage of obtaining pure strains that can be used for further experiments. However, due to the long cultivation time and complex operation method, the traditional cultivation-dependent method has the disadvantages of time-consuming and laborious.

In recent years, with the development of microbiome, targeted amplicon sequencing (16S rRNA gene of prokaryotes). The 18S rRNA and the Internal transcriptional spacer [ITS] gene of eukaryotes ushered in a new era of microbiome research, By clustering sequences, OTU division and comparison in databases (bacteria and archaea 16S rRNA database, fungi 18S rRNA database, fungi ITS database and functional gene database), the understanding of microbial composition and function of Daqu has been improved (Barooah et al., 2022). In addition, more advanced omics techniques such as metagenomics (which can not only provide insights into taxonomic classifications of the Daqu sequence at the species level, especially for the identification of culture-independent microorganisms, but also reveal details of microbiome assembly and underlying gene function) (Yang et al., 2021), Metatranscriptomics (can further explain the active microbial composition and enzyme profile in Daqu based on the overall expression of mRNA in the microbiome) (Song et al., 2017), Metaproteomics (which can detect changes in microbiome protein expression in Daqu and identify key enzyme-producing microbiome; Xia et al., 2022) further provides useful information on the activity and function of the microbiome in Daqu. But there is no doubt that the use of omics requires more financial support and the ability to analyze and mine large-scale data sets.

### 3.2. Traditional culture-dependent screening of functional microorganisms

It has been proved that there are abundant microorganism resources in Daqu and the brewing process (Xu et al., 2017; Wang D. Q. et al., 2019; Chen et al., 2022). The traditional dependent culture method mainly takes the characteristic flavor substances and process links of different flavor types as the guiding ideology to separate and screen the culturable microorganisms in the samples and study their functional characteristics (Zou et al., 2018). A large number of functional microorganisms were identified by screening from Daqu and brewing environment samples (Table 1). Among them, the functional yeasts are mainly *Saccharomyces cerevisiae*, *Wickerhamomyces anomalus*, *Pichia kudriavzevii*, and *Saccharomycopsis fibuligera*. *Wickerhamomyces anomalus* has good aroma and enzyme-producing characteristics and is enriched and screened in a variety of aromatic Baijiu Daqu and brewing environments. In addition, non-dominant yeast, such as *Lodderomyces elongisporus* and *Hyphopichia burtonii*, are unique in Maotai-flavor Baijiu Daqu, which contribute to alcohol production and aroma.

**Table 1:** Isolation, identification, and functional characteristics of Baijiu-brewing functional microorganisms.

Classification of species	Samples	Strains	Characteristic	References
Yeast	Luzhou-flavor Baijiu Daqu	*Wickerhamomyces anomalus*	Metabolites: pyrazine, phenethyl alcohol and guaiacol	Ming et al. (2015)
	Luzhou-flavor Baijiu Daqu	*Wickerhamomyces anomalus*	High yield of ethyl acetate	Fan et al. (2018)
	Luzhou-flavor Baijiu Daqu	*Zygosaccharomyces cidri*	Aroma, high temperature and high alcohol	Dong et al. (2020)
	Luzhou-flavor Baijiu Daqu	*Saccharomyces cerevisiae*	Ability to produce ethanol is strong, but the number of volatile compounds produced is relatively low	Pu et al. (2021)
		*Wickerhamomyces anomalus*	Fermentation efficiency is relatively poor, but produces large amounts of volatile compounds	
	Fen-flavor Baijiu Daqu	*Pichia kudriavzevii*	Not detailed	Li R. Y. et al. (2018)
		*Wickerhamomyces anomalus*	Positive impact on flavor of Daqu derivative products	
		*Saccharomyces cerevisiae*	Advantage: ethanol production	
		*Saccharomycopsis fibuligera*	Advantages: Transform starch or polysaccharide into soluble sugar	
	Maotai-flavor Baijiu Daqu	*Lodderomyces elongisporus*	Producing polyols	Bai et al. (2017)
		*Pichia kudriavzevii*		
	Maotai-flavor Baijiu Daqu	*Saccharomycopsis fibuliqura*	Aroma, alcohol production	Jiang et al. (2017)
		*Saccharomyces cerevisiae*		
		*Lodderomyces elongisporus*		
		*Hyphopichia burtonii*		
		*Pichia farinosa*		
		*Pichia kudriavzeviil*		
	Maotai-flavor Baijiu Daqu	*Saccharomycopsis fibuligera*	Produce flower and fruit flavor	Luo et al. (2016)
	Laobaigan-flavor Baijiu Daqu	*Wickerhamomyces anomalus*	High yield of ethyl acetate by acyltransferase pathway, high tolerance to ethanol and ethyl acetate	Fu et al. (2018)
	Baijiu-brewing environment	*Pichia kudriavzevii*	High yield of 2-phenylethanol	Fan G. S. et al. (2020)
	Daqu	*Corynespora Portugal*	High yield of ethyl caproate, β-phenylethanol and terpenes	Fan G. S. et al. (2021)
	Special-flavor Baijiu Daqu	*S*. *cersvisiae*	High fermenting power	Chen Y. et al. (2021)
		*Wickerhamomyces anomalus*	High esterification and high yield of volatile metabolites	
Bacteria	Luzhou-flavor Baijiu Daqu	*Geobacillus thermoglucosidasius*	High yield esterifying enzyme	Luo et al. (2012)
	Luzhou-flavor Baijiu Daqu	*Bacillus licheniformis*	Aroma-producing functional microorganisms	Ming et al. (2015)
	Luzhou-flavor Baijiu Daqu	*Bacillus cereus*	High yield of ethyl caproate and non-propanol	Zhao et al. (2017)
	Luzhou-flavor Baijiu Daqu	*Staphylococcus epidermidis*	High yield esterifying enzyme	Liu et al. (2021)
	Fen-flavor Baijiu Daqu	*Bacillus licheniformis*	Hydrolyzed starch and polysaccharides	Li R. Y. et al. (2018)
		*Pediococcus pentosaceus*	Not detailed	
		*Lactobacillus plantarum*	Starch dissolution and protein Decomposition activity		
Fen-flavor Baijiu Daqu	*Bacillus licheniformis*	Aroma: ethyl butyrate, 4-ethylguaiacol, 3-hydroxy-2-butanone, phenylacetaldehyde, β-phenylethanol	Liu X. et al. (2022)		
Maotai-flavor Baijiu Daqu	*Bacillus licheniformis*	Production of acetoin, tetramethylpyrazine and furazolidone	Zhang (2009)		
Middle-temperature Daqu	*Bacillus velezensis*	High yield protease	Liu Y. B. et al. (2022)		
High-temperature Daqu	*Bacillus subtilis* YHB0165	High yield tetramethylpyrazine	Zhang C. et al. (2022)		
	*Bacillus subtilis* YHB0169	High yield tetramethylpyrazine			
	Bacillus megatherium YHB0170	High yield tetramethylpyrazin, acetic acid			
	*Bacillus subtilis* YHB0171	High yield tetramethylpyrazine			
Fermented grains of Chen-flavor Baijiu	*Bacillus aryabhattai*	Aroma-producing	Wu et al. (2018)		
	*Bacillus licheniform*	Aroma: 3-hydroxy-2-butanone, 2,3-butanediol, high yield of protease and amylase, optimum growth temperature is 50°C			
	*Bacillus subtilis*	Aroma-producing			
	*Bacillus amyloliquefaciens*	Aroma-producing			
Fermented grains of Luzhou-flavor Baijiu	*Cellulosimicrobium cellulans*	Cellulase, amylase, protease activity under acidic, neutral, alkaline conditions, tolerance to 9% ethanol	Liu et al. (2020)		
Baijiu fermentation raw materials	*Acetobacter*	High yield ferulic acid esterase	Wu et al. (2021)		
Pit mud	*Suwonensis Rummeliibacillus suwonensis*	Coding gene with ester forming	Zhou W. et al. (2022)		
	*Clostridium tyrobutyricum*	Coding gene with ester forming			
	*Lactobacillus buchneri*	Coding gene with ester forming	
Mold	Luzhou-flavor Baijiu Daqu	*Rhizopus arrhizus*	High yield esterifying enzyme	Huang et al. (2012)
	Luzhou-flavor Baijiu Daqu	*Aspergillus clavatus*	High-yield glucoamylase	Ye et al. (2013)
		*Aspergillus niger*	High-yield glucoamylase	
	Maotai-flavor Baijiu Daqu	*Eurotium amsterdamense*	High-yield saccharifying amylase, acid protease, pectinase, lipase and cellulase, volatile flavor substances mainly higher alcohols, ketones and furans	Wang et al. (2016)
	Baijiu starter	*Aspergillus oryzae*	High yield glucoamylase, resistant to 10% ethanol	Liu M. et al. (2018)
	Baijiu starter	*Rhizopus*	Aroma-producing	Zhu et al. (2019)
	Middle-and high-temperature Daqu	*Rhizomucor pusillus*	Heat-resistant, high yield glucoamylase, protease	Li Z. et al. (2020)
	Fermented grains of Maotai-flavor Baijiu	*Paecilomyces variotii*	high-yield amylase	Chen et al. (2014)
		*Aspergillus oryzae*	High-yield α-amylase		
Sesame-flavor Baijiu starter	*Aureobasidium brucei*	Heat-resistant, high yield glucoamylase	Li et al. (2021)		
	MJ10	High-yield glucoamylase			
	MJ2	high yield protease			
	MJ3	High-yield α-amylase			
Baijiu-brewing environment	*Aspergillus tabacinus*	High-yield α-amylase	Lei et al. (2022)		
	*Penicillium limosum*	High yield neutral protease			
	*Scopulariopsis sclerotiorum*	High yield pectinase and cellulase	
Actinomycete	Luzhou-flavor Baijiu Daqu	*Streptomyces lividans*	Producing amylase, protease, pectinase, cellulase, lipase and esterase	Hou et al. (2019)
		*Nocardiopsis*		
		*Streptomyces*		
		*Streptomyces azureus*		
	Maotai-flavor Baijiu Daqu	*Streptomyces cacaoi*	Pectinase producing, tetramethylpyrazine and esters	Luo et al. (2018)
		*Streptomyces zaomyceticus*		
	Maotai-flavor Baijiu Daqu	*Streptomyces bangladeshensis*	Glycosylase and pectinase producing ability is more prominent, ester, terpene, tetramethylpyrazine producing	Wang X. et al. (2018)
	Maotai-flavor Baijiu Daqu	*Laceyella sacchari*	Production of large amounts of pyrazines, aromatic substances	Li D. et al. (2018)
	Maotai-flavor Baijiu Daqu	*Thermoactinomyces vulgaris*	High yield protease	Wang et al. (2022)
	Maotai-flavor Baijiu Daqu	*Streptomyces rochei*	High yield protease	Yu et al. (2017)

The functional bacteria were mainly *Bacillus*. *Bacillus* is one of the important functional bacteria in the fermentation process of Daqu and fermented grains. It can secrete enzymes to hydrolyze starch, protein, pectin and other substrates, promote the smooth fermentation process, and produce health factors and aroma substances such as tetramethylpyrazine.

*Aspergillus* and *Rhizopus* showed higher glucoamylase and α-amylase activities. They are the key functional microorganisms to produce liquor, fragrance and enzyme during Daqu fermentation and Baijiu-brewing, and directly affect the fermentation quality of Daqu and the formation of Baijiu body flavor.

*Actinomycetes* also showed high enzyme production capacity, such as gelase, amylase, protease, which also play a certain role in the formation of aroma. The studies on actinomycetes mainly focus on Maotai-flavor Baijiu Daqu. Due to the high-temperature preparation process, high abundance of high temperature resistant actinomycetes are enriched. According to the fermentation test, actinomycetes showed a certain ability to produce tetramethylpyrazine, which contributed to the formation of the main aroma of Maotai-flavor Baijiu.

However, the microbiota of Daqu and its brewing environment is complex and diverse, and the traditional dependent culture technology can only obtain 0.1–1.0% of the microorganisms in the environment, which greatly limits the study of microbial community diversity, flavor characteristics and formation mechanism of Chinese Baijiu fermentation (Kang et al., 2022). Therefore, the identification of uncultured microorganisms has become an important subject for the in-depth understanding of Daqu and the Baijiu fermentation mechanism.

### 3.3. Study of functional microbiota based on multi-omics linkage

The dominant microflora with higher abundance in the brewing environment is generally considered as the core microbiota (Zhang J. et al., 2022). Traditionally, culture-dependent techniques have been used to isolate and identify species from Baijiu fermentation, and although these studies have shed some light on microbial populations, our understanding of the core functional microbiota in Baijiu fermentation is still limited. With the rapid development of histological technology in recent years, the study of microorganisms in Daqu is no longer limited to traditional culturable techniques, but researchers have used genomics, transcriptomics, proteomics, metabolomics and statistics to study the dominant microbial community, flavor-related microbiota and prevalent microbiota, thus further exploring the core functional microbiota in Daqu fermentation and Baijiu-brewing process (Wang S. L. et al., 2019). Song et al. (2017) combined high-throughput amplicon sequencing (16S rRNA gene amplicon sequencing and internal transcribed space amplicon sequencing), metatranscriptome sequencing technology, ultra-high performance liquid chromatography and headspace solid-phase microextraction-gas chromatography–mass spectrometry to determine the core microbial community of Daqu of Maotai-flavor Baijiu. The first stage of Daqu fermentation involves high-level alcohol (ethanol) production, with *Schizosaccharomyces* as the core functional microorganism. The second stage involves high levels of acid (lactic acid and acetic acid) production, and *Lactobacillus* is the core functional microorganism. Wang S. L. et al. (2019) used Chinese light-flavor Baijiu as a model system, based on its flavor production and symbiotic performance, revealed that the core microflora was *Lactobacillus*, *Saccharomyces*, *Pichia*, *Geotrichum*, and *Candida*. Synthetic microbial consortia capable of reproducing complex flavor metabolism were constructed using *Lactobacillus acetotolerans*, *Pichia kudriavzevii*, *Geotrichum candidum*, *Candida vini*, *Saccharomyces cerevisiae*.

In fact, more and more studies have used the above methods to obtain large-scale omics data sets and extended the research strategy of the Baijiu-brewing mechanism through gene function enrichment analysis and correlation analysis. Table 2 lists the research status of functional microbial composition, characteristics, and research strategies of some important samples of Daqu, pit mud, and fermented grains.

**Table 2:** Composition, functional characteristics, and research methods of core functional microbiota.

Samples	Research methods	Functional microorganisms	Characteristic	References
Luzhou-flavor Baijiu Daqu	Pyrophosphate sequencing HPLC HS-SPME-GC–MS	Saccharopolyspora *Rhizomucor*	Volatile metabolite formation related	Deng et al. (2021)
Luzhou-flavor Baijiu Daqu and Maotai-flavor Baijiu Daqu	Metaproteomics	*Kroppenstedtia*、*Lichtheimia*、*Byssochlamys*、*Thermomyces*、*Thermoascu*	*Thermoascu* contributed the most to cellulase system, others to α-amylase, glucoamylase and α-glucosidase	Xia et al. (2022)
High-temperature Daqu	Amplifier sequencing Physicochemical analysis Metabolomics	*Kroppenstedtia*，*Virgibacillus*, *Scopulibacillus*、*Staphylococcus*、*Thermoascus*、*Aspergillus*	Positively correlated with almost all rich volatiles	Shi et al. (2022)
Middle-temperature Daqu	Metagenomics Physicochemical analysis HS-SPME-GC–MS	Bacteria: Lactobacillus (mainly *Weissella*, *Lactobacillus*, and *Pediococcus*) Mildew: Mucor (mainly *Lichtheimia*) and Eurotiales (mainly *Aspergillus*, *Rasamonia* and *Byssochlamys*)	It is responsible for the production of lyases and flavor precursors / compounds in medium-temperature Daqu. The catabolic activities of *Lactobacillus* and Lichtheimia may contribute to the effective self-cultivation of the microbiota	Yang et al. (2021)
Daqu and wheatqu	Whole genome sequencing GC–MS HPLC	*R*. *emersonii*、*W*. *paramesenteroides*, *Leuconostoc citreum*, *Leuconostoc mesenteroides*, *Weissella cibaria* and *P*. *pentosaceus*	Synthesis of flavor compounds	Zhang J. et al. (2022)
Daqu	Third generation single molecule real-time (SMRT) sequencing	*Coccidioides*, *Aspergillus* and *Streptomyces*	Involvement in the metabolic pathways of Daqu fermentation (gluconeogenesis I, sucrose degradation III, pentose phosphate pathway, 5-aminoimidazole ribonucleotide biosynthesis I, methyl ketone biosynthesis, and GDP-mannose biosynthesis)	Zhang Y. D. et al. (2022)
Daqu	Amplifier sequencing Metagenomics HS-SPME-GC–MS	*Kroppenstedtia*、*Thermoactinomyces*、*Bacillus*、*Acinetobacter*、*Brevibacterium*、*Saccharopolyspora*、*Ochrobactrum*、*Aspergillus*、*Byssochlamys*、*Thermoascus* and *Thermomyces*	Production of key enzymes (α-amylase, glucoamylase and protease) and flavor compounds	Zhu et al. (2022)
Daqu	Amplifier sequencing Metaproteomics	*Rhizopus*, *Pichia*, *Wickerhamomyces*, *Saccharomycopsis*, *Aspergillus* and *Saccharomyces*	Coding enzymes and other precursors	Ban et al. (2022)
Fen-flavor Baijiu Daqu	Metagenomics Amplifier sequencing ^1^H NMR spectrum HS-SPME-GC–MS	Bacteria: *Lactobacillus* Yeast: *Saccharomyces cerevisiae*	Has the function of producing ethanol and flavor compounds, and can form a mixture of species biofilm, survive in the starter community, while coexisting with other microorganisms	Fan Y. et al. (2020)
Fen-flavor Baijiu Daqu	Metagenomics Physicochemical analysis	*Bacillus licheniformis*	The most abundant microorganisms at the species level	Hou et al. (2022)
Fermented grains of Fent-flavor Baijiu	Metatranscriptomics Amplifier sequencing	*Saccharomyces*, *Lactobacillus*, *Wickerhamomyces*, *Pediococcus*, *Candida* and *Fecalibacterium*	Active microorganisms involved in carbohydrate hydrolysis, ethanol production and flavor generation	Pan et al. (2022)
Pit mud	Metagenomics	*Syntrophomonas*, *Thermacetogenium*, *Clostridium*, *Methanobacterium*, *Methanoculleus* and *Methanosarcina*, *Proteniphilum*, *Prevotella*, *Chloroflexi*	Containing most of the metabolic potential genes, *Chloroflexi* may be involved in the production of caproic acid (CA)	Fu et al. (2021)

From the perspective of research methods, the characteristics of functional microbiomes determined by different combinations of techniques are not consistent. Most studies tend to establish an association network between the microbial community and flavor compounds, so as to define the core aromatic functional microflora; On this basis, further analysis of gene function from the DNA level, analysis of active microorganisms and enzyme profile from the RNA level, analysis of protein expression and enzyme producing microorganisms from the protein level can fully determine the core enzyme producing microorganisms in Daqu.

In addition, the functional microbiota composition of different types of Daqu samples was different. As mentioned earlier, Daqu can be classified according to fermentation temperature, flavor characteristics, etc. High temperature resistant bacteria such as *Kroppenstedtia*, *Thermomyces*, *Thermoascus* are mainly present in High-temperature Daqu or Maotai-flavor Baijiu Daqu; While *Saccharomyces*, *Weissella*, *Lactobacillus* and *Pediococcus* are the main functional bacteria in Middle-temperature Daqu and Fen-flavor Baijiu Daqu. We also noticed that the composition and species of core functional microorganisms in Fen-flavor Baijiu Daqu were similar to those in fermented grains of Fen-flavor Baijiu. The fermentation method is ground tank fermentation, and the microorganism mainly comes from Daqu, so the functional microbiome has certain similarities. We speculate that the functional microbial differences between Luzhou-flavor and Maotai-flavor Baijiu Daqu and fermented grains may be greater because the microbial sources of fermented grains of these two types are more extensive. For example, fermented grains of Luzhou-flavor Baijiu fermented in the cellar, pit mud microorganisms will participate in the fermentation, some methanogens (*Methanobacterium*, *Methanoculleus* and *Methanosarcina*) and *Chloroflexi* involved in the formation of caproic acid and ethyl caproate. Maotai-flavor Baijiu brewing process of high-temperature accumulation, environmental microorganisms will be further enriched.

## 4. Application strategy of functional microorganisms

**Figure 2:**
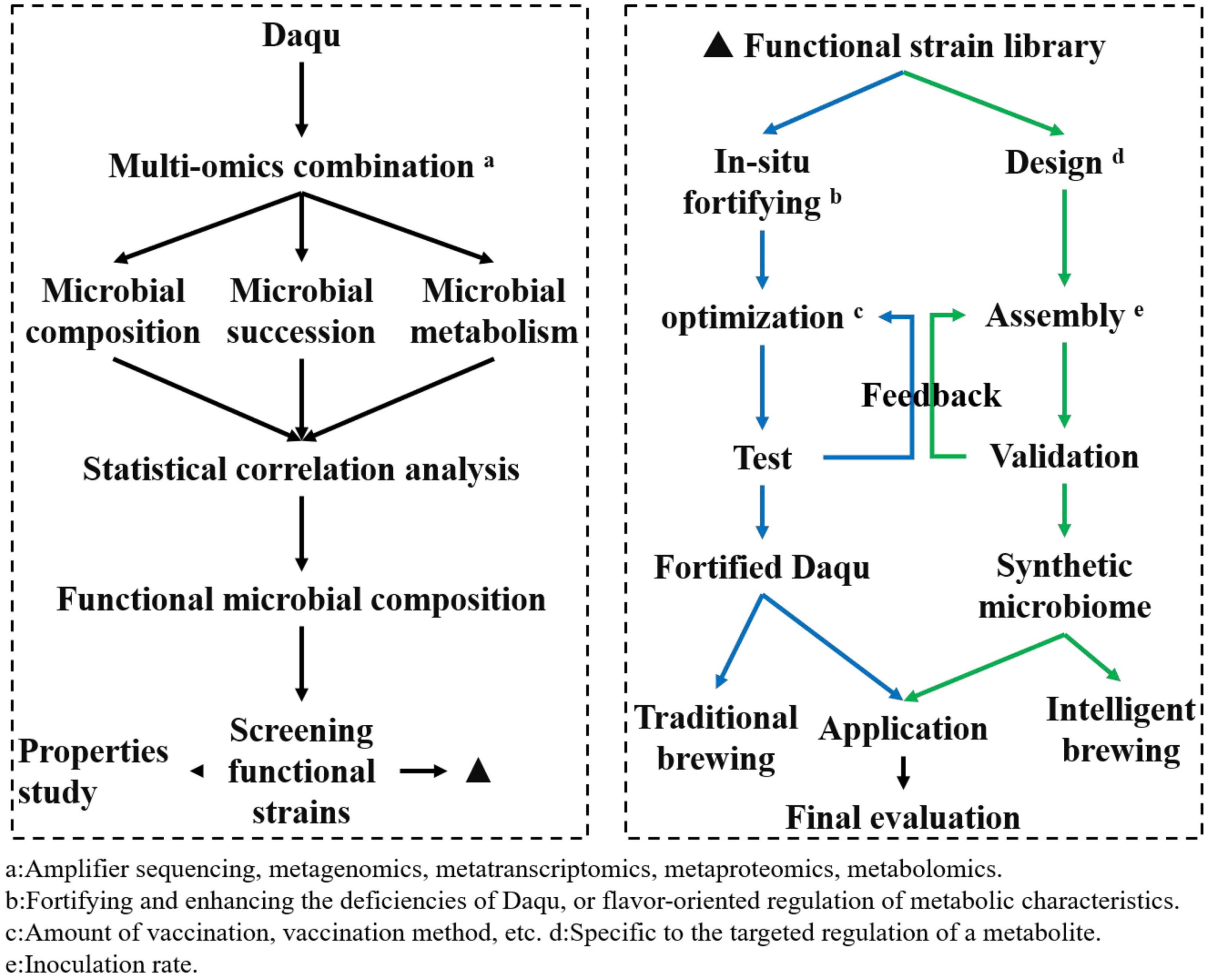
Application strategy of Baijiu-making functional microorganisms.

Determining the composition, succession law, and fermentation characteristics of core functional microorganisms in the fermentation process of Daqu is conducive to guiding the breeding of functional microorganisms, and further studying the interaction mechanism between strains based on breeding, laying a foundation for strengthening functional strains. Ecological fermentation indicates that adjusting the initial microbial community can reconstruct the fermented microbial community, recombine the core functional microbiota community, and control the fermentation process to control the community to efficiently produce the target product, achieving microbial community regulation through a top-down approach (Du R. et al., 2021). Besides, the identification of core microbiota is conducive to the construction of a synthetic microbiota. Simplified microbial flora is an effective way of directional regulation, which is of great significance for the directional, stable, and safe production of Daqu (Wang S. L. et al., 2019). The functional microorganism’s application strategy is shown in Figure 2.

In the first step, the microbial structure, succession law and metabolic characteristics of Daqu fermentation were analyzed, and the composition of functional microbial groups was determined by multi-omics combination combined with statistical correlation analysis (each omics technology has been introduced in section 3); on the one hand, to understand the mechanism of Daqu fermentation changes, provide a theoretical basis for the application of functional microorganisms; on the other hand, the composition and function of functional microorganisms are investigated to provide information support for screening functional microorganisms. The second step is to establish a functional strain library based on the screened functional microorganisms; the fermentation characteristics and safety of the screened strains were evaluated, and a strain characteristic classification information database was established for the subsequent selection of functional microbial strains. The third step is to construct *in situ* fortified microbial communities and synthetic microbial communities based on functional microorganisms; for functional microbial fortified Daqu, the inoculation method and inoculum number of functional microorganisms will be optimized and tested according to the need; for synthetic microbial communities, the composition and inoculation ratio will be determined and verified. This process is the most complex and challenging part of the application of functional microorganisms, the main premise and direction of the application of functional microorganisms are positive; then the existing problem is how to ensure the normal fermentation of Daqu under bioturbation and can achieve the purpose of fortification. If it is a synthetic microbiota, which microorganisms need to be combined, how to control the ratio of each strain, and whether the flavor characteristics and taste of Baijiu can be reproduced by using synthetic microbiota, these questions are still urgent to be solved. In the fourth step, evaluation of the application of fortified Daqu and synthetic microbiota; in actual production, Baijiu brewing is carried out using fortified Daqu and bacteriological agents constructed with synthetic microbiota, and the fermentation process (fermentation quality, raw material utilization rate, etc) and the quality of raw Baijiu (yield, flavor characteristics, taste, etc) are evaluated.

## 5. Functional microorganisms fortification

### 5.1. Fortification of functional yeast strains

Yeast is a kind of single-cell fungus, which is widely used in food, the chemical industry, medicine, and agriculture. Yeast is a very important microorganism in the Baijiu-brewing industry. Without yeast, there is no alcoholic beverage. Yeast mainly conducts alcoholic fermentation and produces aroma in Baijiu production, which is of great significance to Baijiu flavor and quality (Wang D. Q. et al., 2019).

*Saccharomyces cerevisiae*, *Wickerhamomyces anomalus*, *Saccharomycopsis fibuligera*, *Pichia caribbica*, and *Pichia kudriavzeviia* are the main yeasts in Daqu (Zheng et al., 2011; Wang D. Q. et al., 2019; Zhou Q. et al., 2022). In fact, Baijiu is particularly focused on the composition and content of its flavor, so the yeast fortification is mostly oriented towards producing high quality flavor. It was reported that the fortification application of *Saccharomyces cerevisiae* with high ethyl caproate yield increased the ethyl caproate content (Li W. W. et al., 2020). The use of high-yielding ester Saccharomyces cerevisiae fortification of the production of the Daqu not only increased the ester content, and can reduce the alcohol and aldehyde content, the content of beneficial aroma components of the Daqu partially enhanced (Jiang, 2017). However, subsequent studies found that *Saccharomyces cerevisiae* was the most capable of producing ethanol through fermentation but produced relatively less volatile compounds, while *Wickerhamomyces anomalus* was relatively less efficient in fermentation but produced the greatest amount of volatile compounds (Pu et al., 2021). Therefore, *Wickerhamomyces anomalus* is used more often to enhance the quality of Baijiu Daqu and Baijiu. Hong et al. (2021) replaced the traditional Daqu with functional microbial fortification with different ester-producing yeasts as variables to simulate the solid-state fermentation of Special-flavor Baijiu. *Wickerhamomyces anomalus* fortification increased the ethanol content in the fermented grains to 3.67% v/v and volatile matter content to 136.02 mg/kg, and fortification did not change the dominant bacterial genus relative abundance of *Lactobacillus*. Xu et al. (2021) isolated and purified the aroma-producing *Wickerhamomyces anomalus* Y87 from Luzhou-flavor Baijiu Daqu. It was used to strengthen the production of Daqu and Baijiu-brewing. The content of ethyl acetate in Baijiu increased by 36.6%, the content of fusel alcohol decreased by 7.1%, and the content of n-propanol decreased by 16.1%. The Baijiu had the typical characteristics of Light-flavor Xiaoqu Baijiu, with a mellow and sweet entrance and pure fragrance. In addition, according to the production requirements, different yeasts can be combined in different proportions to strengthen together. Pu et al. (2021) reported that two yeast strains, *Saccharomyces cerevisiae*, and *Wickerhamomyces anomalus*, were used in mixed culture to produce fortified Daqu, and when a 2% inoculum consisting of a 1:2 (v/v) ratio was used, greatest fermentation, saccharification, liquefaction, and esterification capacities were obtained as well as high levels of volatile compounds, and when the produced Baijiu was compared with the unfortified control Daqu, significantly higher levels of flavor compounds were found after fortification. Meanwhile, some low-abundance yeasts play an important role in flavor production, such as *Clavispora lusitaniae* fortified with increased ethyl caproate content (Li W. W. et al., 2020). The strengthening of *Candida* Y18 increased the relative abundance of total alcohols and total esters in fermented grains. The relative abundance of ethanol, isoamyl acetate, and ethyl acetate increased to a certain extent, and the aroma components of isoborneol ethanol, isobutanol, methyl benzoate, 2-phenylethyl acetate, and phenyl caproate were metabolized (Xing et al., 2018).

### 5.2. Fortification of functional bacterial strains

The whole enzyme production process of bacteria will directly affect the fermentation quality of Daqu and the brewing quality of Baijiu and is usually reflected in its protease, amylase, esterification enzyme, and other different types of enzyme characteristics, and produce rich flavor substances and its precursors. *Bacillus* is a kind of aerobic or facultative anaerobic bacillus, which can produce spores. *Bacillus* is widely distributed in the process of Baijiu-brewing and has high diversity, such as *Bacillus licheniformis*, *Bacillus amyloliquefaciens*, *Bacillus subtilis*, *Bacillus tarda*, *Paenibacillus*, *Bacillus cereus*, *Bacillus pumilus*, *Bacillus methylotrophicus*, etc.

Fortification of Daqu has a positive effect on the structure of microbial communities and the content of aroma components and the storage period of Daqu (Yan, 2012; Li et al., 2021). The fortification of *Bacillus licheniformis* significantly affected the physicochemical properties, microbial community diversity, and volatile fractions of the Luzhou-flavor Baijiu Daqu, with pyrazine and alcohol contents increasing by 5074.49 and 440.50%, respectively, and the fortification of Maotai-flavor Baijiu Daqu helped highlight the stylistic characteristics of its (Hu, 2016; Chen X. et al., 2021). The combination of *Bacillus licheniformis* and *Bacillus velezensis* fortification altered the relative abundance of 22 genera in the four layers (surface, fire ring, middle, and core) of the Daqu, with an increase in the abundance of *Bacillus* and *Aspergillus* and a decrease in the abundance of *Thermoascomycetes*, *Lactobacillus*, and some other genera, and a significant increase in the esterification capacity of the fire ring and surface layer and an increase in the content of alcohols, acids, and ketones (except esters) after inoculation (Xu et al., 2022). High 4-ethyl guaiacol (4-EG) producing strain *Bacillus subtilis* D-31, and tetramethylpyrazine (TTMP) producing strain *Bacillus velezensis* M-14 fortification, respectively, showed that D-31 fortification showed an increase in pyrazines, TTMP, esters and 4-EG compared to the control Daqu by 117.00, 130.54, 175.34, and 266.20%, respectively, while the results of M-14 fortification were 89.26, 149.44, 355.63, and 501.40%, and the pyrazines, TTMP, esters, and 4-EG of the co-cultivated Daqu fortification of the two bacteria combinations increased by 22.60, 16.74, 83.46, and 323.79%, while the co-cultivation of the fortified Daqu increased the saccharification and liquefaction power, and the content of volatile components increased (Wu et al., 2017). Functional microbial fortification has certain bioturbation effects and interferes with enzyme-encoded gene expression. The enrichment of *Bacillus velezensis* and *Bacillus subtilis* increased the abundance of *Bacillus*, *Lactobacillus*, and *Candida albicans*, and significantly improved the liquefaction ability, saccharification ability, and esterification ability. In addition, many enzymes involved in saccharification, ethanol fermentation, and aroma formation were predicted, and the abundance of their coding genes was significantly increased in fortified Daqu (He G. et al., 2019). *B*. *velezensis* and *B*. *subtilis* were jointly fortified with Daqu applied to the fermentation of fermented grains in different spatial locations, with varying degrees of influence on the microbial community structure and volatile aroma substances of the fermented grains, and with a high content of Baijiu skeletal flavor components, mainly including important esters and aromatic compounds (He G. Q. et al., 2019). Overall, bioaugmentation of inoculated *B*. *velezensis* and *B*. *subtilis* altered the microbial community to regulate their metabolic activities.

### 5.3. Fortification of functional mold strains

Molds in Daqu can not only form hyphae to penetrate the entire Daqu embryo to ensure the smooth discharge of moisture so as to avoid rancidity of Daqu, but also give Daqu saccharification, liquefaction, protein decomposition, and other hydrolysis ability and esterification ability, the formation of Baijiu style played an important role. It has been reported that the fortification of *Eurotium cristatum* has different effects on the number of microbial groups (molds, yeasts, bacteria and *Eurotium cristatum*), conventional physicochemical indicators (cultivation temperature, moisture, acidity and starch content) and enzymatic activity (fermentation power, liquefaction enzyme activity and glycolytic enzyme activity; Fan B. et al., 2021). Mixed fermentation of *Monascus*, *Aspergillus oryzae* and *Rhizopus* increased Baijiu yield and enriched flavor substances (Ma, 2022). The contents of ethyl propionate and ethyl 2-methylbutyrate in *Paecilomyces variotii* PV3 fortified Daqu increased significantly (Deng, 2022).

However, there are few reports on the use of mold strengthening. The potential influencing factors may be that the strengthening of mold destroys the steady state of Daqu microecology or fermented grains microecology. First, excessive proliferation of mold often leads to significant substrate hardening, affecting the Daqu and fermented grains fermentation process (Zhang et al., 2021); Second, the growth of filamentous fungi will produce secondary metabolites that affect microbial growth, such as resulting in the inhibition of ethanol metabolism of yeast (Wei et al., 2021); Third, the fortification of mold leads to greater saccharification power and rapid accumulation of reducing sugar content, which inhibits the growth and metabolism of yeast and disrupts the balance of bilateral fermentation of Daqu and fermented grains, resulting in some adverse effects (Zhang et al., 2015).

### 5.4. Multi-strain fortification and synthesis microbiota

In order to further analyze the interrelationship between functional microorganisms in the original ecological site, and to deepen the understanding of functional microorganism utilization, the researchers also conducted an investigation experiment on the effect of multiple functional strains of combination fortification of the Daqu. The study showed that the combination of multiple functional microorganisms’ fortification had a small perturbation effect on the composition and succession of the Daqu microbiota, and there was no significant difference between the microbiota in traditional Daqu and bio-enhanced Daqu (Li et al., 2017).

In addition, multi-strain fortification does not significantly affect the fermentation process, and can also improve the quality of the Daqu and Baijiu. Zhou et al. (2013) comprehensively utilized dozens of beneficial functional microorganisms in a variety of ways to fortify the Daqu in Baijiu production. Compared with the traditional Daqu, the mycelium of the fortified Daqu is fuller, the flavor of the Daqu is more prominent and richer, and the temperature change of the cellar using the fortified Daqu is fully in line with the best state of before slow, medium strong, and after slow down. Zeng (2016) obtained the fortified Daqu with the mixed ratio of 18: 6: 1 of functional microbial bacteria, molds and yeasts in the Daqu of Maotai-flavor Baijiu. The skin of the fortified Daqu was grayish white with brown color, the aroma was prominent, the section was covered with white spotted hyphae, and there was no foreign matter. The workshop application test showed that the Maotai-flavor of Baijiu was more prominent, the burnt aroma and paste aroma were more coordinated, the entrance was relatively smooth, the Baijiu body was full, the aftertaste was longer and slightly astringent, and the quality of the seventh round of Baijiu was improved. Liu et al. (2019) strengthened Maotai-flavor Daqu by optimizing the combination of 5 strains of functional microorganisms, and applied it to the production of Maotai-flavor Baijiu. The results showed that the flavor of Maotai-flavor Daqu was strong by adding an appropriate amount of functional microorganisms, and the flavor of Maotai-flavor Baijiu, flavor retention in empty cups, fineness and typicality were significantly improved. Wang (2017) constructed a composite functional microbial agent with *Schizosaccharomyces pombe*: *Issatchenkia orientalis*: *Bacillus licheniformis* + *Rhizopus* (10^6^: 10^5^: 10^5^ + 1%), which was added to different months of Daqu or replaced different proportions of 4-month Daqu. The results showed that this group of functional microbial agents had the effect of increasing the yield of fermented ethanol and reducing the content of n-propanol, and had the function of improving the yield and quality of Baijiu.

As mentioned before (section 5.3) filamentous fungi have some instability in single fungal fortification applications, similarly in multi-strain combination fortification the growth characteristics of the fungus and the interactions with other microorganisms should be fully considered. Huo (2019) used *filamentous fungus* FBKL 3.0009, *Pichia* FBKL 2.0008 and *Bacillus licheniformis* FBKL 1.0199 to make fortified Daqu. Compared with the fortified Daqu without adding mold, the saccharifying power of the fortified Daqu was improved, and the saccharifying power of the fortified Daqu with more mold was significantly improved. This indicated that the addition of mold promoted the saccharifying power of the fortified Daqu, but the addition of mold inhibited the production of pyrazines.

Currently, the conversion of traditional fermented foods, including bread, wine, chocolate, pickles and wine from natural fermentation to controlled synthetic microbial fermentation is essential to achieve production stability of fermented foods. Compared with the above-mentioned traditional fermented foods, the research on synthetic microbiota in the brewing process of Baijiu is in its initial stage. On the one hand, the composition of the core functional microbiota in the Baijiu brewing process is not fully understood, the mechanism of microbial interactions is still not completely clear, and there are still some technical difficulties that need to be broken through as to how the synthetic microbiota can best fit the functions and roles of the Daqu microflora. On the other hand, synthetic microbiota replaces the role of Daqu (Daqu provides microorganisms, enzymes and substances for Baijiu fermentation), which is a newly emerged technology compared with traditional Baijiu fermentation, and innovates the Baijiu brewing process, but there is still a certain distance in a production application. Wang S. L. et al. (2019) used the fermentation of Chinese Light-flavor (Fen-flavor) Baijiu as a model system and used *Lactobacillus*, *Saccharomyces*, *Pichia*, *Geotrichum*, and *Candida* to construct a synthetic microbial community instead of Daqu as a starter. The synthetic core community produced 77.27% of the flavor compounds and showed a dynamic distribution similar to the natural Baijiu fermentation process.

In summary, the purposeful application of functional strains to Daqu fermentation can improve the performance of Daqu and improve Daqu flavor substances, and application to brewing production can increase the Baijiu yield and improve Baijiu quality. Based on the simplified synthetic microbiota applied to Baijiu brewing can achieve the reproduction of flavor metabolism to a certain extent.

## 6. Conclusion and prospect

The production process of Chinese Baijiu is extensive and multi-bacteria and multi-enzyme mixed fermentation. In addition to the application of the strains introduced above to the fortification and production of Daqu, there are still many strains that can be isolated and cultured and many strains that have not been able to be isolated in the fermentation process of Baijiu. It plays a metabolic regulation and other important roles, but the mechanism and conditions of action have not been fully explained due to the limitations of existing methods and research methods, which is not conducive to the stability of Baijiu quality and flavor regulation. Therefore, the core microflora formed by flavor compounds and the key factors affecting the core microflora needs to be further explored and explored. In addition, the culturability of uncultured functional microorganisms based on omics technology is an urgent problem to be solved in further research.

Daqu fermentation has strong environmental dependence, and there are many kinds of microorganisms. Different flavors of Baijiu have regional differences and brewing process differences. At the same time, a different flavor of Baijiu has different requirements for the flavor characteristics of the final Baijiu. Therefore, it is necessary to accurately determine the core functional microorganisms in the fermentation process of Daqu and fermented grains. Based on microbial analysis technology and metabolomics, the microbial community structure, succession law, and metabolic characteristics of the Daqu fermentation process were analyzed, and the research route of core microorganisms was determined by statistical analysis. It proved to be feasible. Although strains with high alcohol, enzyme, and specific flavor substances can be explored, Daqu fermentation and fermented grains fermentation are highly complex microecological interaction systems. Whether the fortified functional strains can have certain abundance and advantages in the fermentation system, whether they can maintain their high alcohol, enzyme, and aroma characteristics, and whether they can improve the quality of Daqu and Baijiu still need to be effectively verified.

Using functional strains to make fortified Daqu is to use its functional characteristics to change the quality of Daqu fermentation. The essence is that the proportion of initial microbial community inoculated with Daqu has changed, which in turn changes the interaction of microbial succession. Therefore, before fortifying functional microorganisms, it is necessary to further clarify the characteristics of interactions between microorganisms during Daqu fermentation.

On the basis of strengthened applied research the designed synthetic flora can be further utilized for flavor regulation and control in Daqu fermentation and fermented grains fermentation. However, in the future, there is a need to regulate the synthetic microbiota from various aspects such as community composition, community regulation, and metabolite regulation, which in turn will lead to the control and standardization of Baijiu quality.

## Author contributions

HL: conceptualization, investigation, visualization, writing-original draft, and writing-review and editing. SL: writing-review and editing. YL: writing-review and editing, investigation, resources, and supervision. MH: writing-review and editing, investigation, resources, and supervision. CP: conceptualization, visualization, writing-review and editing, resources, and supervision. All authors contributed to the article and approved the submitted version.

## Funding

This work supported by the Key Technologies Research and Development Program of Henan Province of China (No. 202102110130), Major Science and Technology Projects of Henan Province of China (No. 181100211400), and Food Science and Engineering Key Discipline Construction Project of Henan University of Animal Husbandry and Economy (No. XJXK202203).

## Conflict of interest

The authors declare that the research was conducted in the absence of any commercial or financial relationships that could be construed as a potential conflict of interest.

## Publisher’s note

All claims expressed in this article are solely those of the authors and do not necessarily represent those of their affiliated organizations, or those of the publisher, the editors and the reviewers. Any product that may be evaluated in this article, or claim that may be made by its manufacturer, is not guaranteed or endorsed by the publisher.
